# Multimorbidity, Mental Illness, and Quality of Care: Preventable Hospitalizations among Medicare Beneficiaries

**DOI:** 10.1155/2012/823294

**Published:** 2012-12-20

**Authors:** Mayank Ajmera, Tricia Lee Wilkins, Patricia A. Findley, Usha Sambamoorthi

**Affiliations:** ^1^Department of Pharmaceutical Systems and Policy, School of Pharmacy, West Virginia University, Morgantown, WV 26506, USA; ^2^School of Social Work, Rutgers University, New Brunswick, NJ 08901, USA; ^3^HSR&D Center for Healthcare Knowledge Management, Veterans Administration New Jersey Healthcare System East Orange, NJ 07018, USA

## Abstract

*Background*. Individuals with multimorbidity are vulnerable to poor quality of care due to issues related to care coordination. Ambulatory care sensitive hospitalizations (ACSHs) are widely accepted quality indicators because they can be avoided by timely, appropriate, and high-quality outpatient care. *Objective*. To examine the association between multimorbidity, mental illness, and ACSH. *Study Design*. We used a longitudinal panel design with data from multiple years (2000–2005) of Medicare Current Beneficiary Survey. Individuals were categorized into three groups: (1) multimorbidity with mental illness (MM/MI); (2) MM/no MI; (3) no MM. Multivariable logistic regressions were used to analyze the association between multimorbidity and ACSH. *Results*. Any ACSH rates varied from 10.8% in MM/MI group to 8.8% in MM/No MI group. Likelihood of any ACSH was higher among beneficiaries with MM/MI (AOR = 1.62; 95% CI = 1.14, 2.30) and MM (AOR = 1.54; 95% CI = 1.12, 2.11) compared to beneficiaries without multimorbidity. There was no statistically significant difference in likelihood of ACSH between MM/MI and MM/No MI groups. *Conclusion*. Multimorbidity (with or without MI) had an independent and significant association with any ACSH. However, presence of mental illness alone was not associated with poor quality of care as measured by ACSH.

## 1. Introduction

Multimorbidity is often defined as the concurrent presence of multiple chronic conditions [1–4]. The prevalence of multimorbidity is highest among the elderly, with estimated rates ranging from 55% to 80% [5, 6]. With advancements in medical technology and prolonged life expectancy, approximately 81 million Americans will be living with multimorbidity by 2020 [7, 8]. Studies have reported adverse health outcomes [9–15], compromised quality of care [16–20], and challenges in disease management, [21] in individuals with multimorbidity. In this context, cooccurring physical and mental illness has been an emerging research area in multimorbidity. High prevalence of cooccurring depression in many chronic physical illnesses and its negative impact on healthcare management has been extensively documented [22–24]. However, research on the concurrent presence of mental illness within a cluster of chronic physical conditions has been sparse. Indeed, from a recent systematic review of 194 articles describing 17 multimorbidity measures, none of the studies distinguished between concurrent presence of mental and physical conditions [25].

Healthcare management in individuals with cooccurring mental and chronic physical conditions can be especially challenging within the USA healthcare system due to fragmentation of care [26–29]. Such fragmentation of care can produce poor quality of care and clinical and economic outcomes. Recent multimorbidity pattern analyses conducted by the Center for Healthcare Strategies revealed that Medicaid beneficiaries with cooccurring mental and chronic physical conditions have significantly higher healthcare expenditures as compared to beneficiaries having only chronic physical conditions [30, 31]. Specifically, one of the top five diagnostic triads among the most expensive 5% of Medicaid beneficiaries includes those with “cardiovascular-pulmonary-psychiatric” conditions [31].

With respect to quality of care, studies have produced inconsistent conclusions regarding the impact of mental illness. Among veteran clinic users with diabetes, quality of care in terms of processes of care such as foot, eye, and HbA1c testing was found to be similar for individuals with and without mental illness [32]. However, other studies have suggested that receipt of quality care was poor for individuals with diabetes and mental illness compared to those without mental illness [33, 34]. Conversely, presence of chronic physical conditions can influence the management of mental illness due to competing demands from physical conditions [35, 36].

Quality of care in terms of hospital admissions has been studied in individuals with multimorbidity [9]. Of special interest are hospitalizations for ambulatory care sensitive conditions because these are established measures of high-quality care. Ambulatory care sensitive hospitalizations (ACSH-) are hospitalizations which could have been avoided by timely, appropriate, and high-quality outpatient care [37]. In 1999, among elderly Medicare beneficiaries, those with multimorbidity (defined as individuals with 4 or more conditions) were 98 times (95% Confidence Interval; 86.11–112.72) as likely to have ACSH as those without any chronic condition [9]. Increased rates of ACSH have been linked with poor access to high-quality outpatient care. Several studies have used ACSH as a quality of care indicator in primary care as well as for indicator for chronic disease management [38–40]. However, to date, no study has examined ACSH as a quality of care indicator among individuals with cooccurring mental illness and chronic physical conditions. 

Therefore, the primary objective of this study was to evaluate the association between multimorbidity with and without mental illness and quality of care as measured by ACSH among Medicare beneficiaries. For this paper we used longitudinal data from a nationally representative survey of Medicare beneficiaries, the Medicare Current Beneficiary Survey (MCBS).

## 2. Methods

### 2.1. Study Design

We used longitudinal panel design to analyze the association between multimorbidity and ACSH using Medicare Current Beneficiary Survey (MCBS), which is a “nationally representative sample of aged, disabled, and noninstitutionalized Medicare beneficiaries” with Medicare claims [41].

### 2.2. Data

The MCBS is a longitudinal, multipurpose survey, representative of Medicare population. The data is continually collected for three rounds per year at four months interval. All the Medicare beneficiaries are followed for three years. MCBS follows a multistage stratified random list sampling design with rotating panel survey design where each panel is followed for 12 interviews. The MCBS data has been used by researchers and policy makers to evaluate the Medicare program and crafting policy for improvement of healthcare for the Medicare population such as shaping of 2003 Medicare Prescription Drug, Improvement, and Modernization Act [42].

The MCBS consists of two modules: (i) “access to care;” (ii) “cost-and use.” Information relating to respondents' access to medical providers and their satisfaction with healthcare for beneficiaries who were enrolled in Medicare for the entire year is present in the access to care module, whereas the “cost and use” files contain information on personal healthcare expenditures and payment sources for all beneficiaries who were eligible for Medicare at any time throughout the calendar year. Expenditures and utilization information regarding healthcare services were obtained from personal interviews conducted every four months as well as Medicare claims. The two data files can be used separately or (for those enrolled for the full year) in combination. The current project utilized multiple years of cost and used files linked with Medicare administrative claims data.

### 2.3. Analytical Sample

The analytical sample was restricted to include community dwelling fee-for-service Medicare beneficiaries who were followed for all 3 years. Additional exclusion criteria were (i) those without any chronic conditions (arthritis, cancer diabetes, heart diseases, hypertension, respiratory diseases, and osteoporosis), (ii) those with mental illness and one physical condition; (iii) very few individuals with no information on self-reported chronic conditions. 

### 2.4. Longitudinal Panels

Using the merged data from multiple years (2000–2005) of the MCBS, four longitudinal panels were created: (i) 2000–2002 (Panel I); (ii) 2001–2003 (Panel II); (iii) 2002–2004 (Panel III); (iv) 2003–2005 (Panel IV). The first year of each panel was used to determine the baseline characteristics and multimorbidity categories, and the two follow-up years were used to determine any ACSH. Each panel contains a nationally representative sample of beneficiaries who were interviewed 12 times (every 4 months) to collect 3 complete years of utilization data. Every panel includes unique beneficiaries not represented in other panels. All the variables included in our analysis were coded consistently across all years.

To arrive at these panels, we only included those who were first interviewed in the year of observation and met our study eligibility criteria. For example, 4,547 were first interviewed in 2000, of which 4,112 had at least one chronic condition. Of these, 3,290 individuals had information for all three years, we excluded 384 institutionalized individuals and 589 HMO enrolled beneficiaries. Finally after eliminating those (*n* = 73) with single chronic physical condition and mental illness we had 2,244 individuals in panel I (*n* = 2, 244). Of the 12,864 individuals interviewed in the year 2001, 4,471 were interviewed for the first time, of which 3,615 were followed for 3 years of interviewing. The final analytic sample after pooling panels obtained after applying similar eligibility criteria consisted of 8,963 beneficiaries, with similar distribution across panels: 2000–2002 (*n* = 2, 244); 2001–2003 (*n* = 2, 260); 2002–2004 (*n* = 2, 217); 2003–2005 (*n* = 2, 242).

### 2.5. Key Independent Variable: Multimorbidity

Based on published studies establishing clinical burden (morbidity and mortality), economic burden (cost and expenditures), and high prevalence, we identified seven chronic physical conditions of priority, from the survey responses recorded in the health status and functioning files of the MCBS [43–46]. These seven chronic physical conditions include arthritis, cancer diabetes, heart diseases (myocardial infarction, coronary heart disease, and other heart conditions), hypertension, respiratory diseases (chronic obstructive pulmonary disease (COPD), and asthma), and osteoporosis [44]. Similarly, presence of any mental illness was assessed based on the self-reported data. The key independent variable was categorized into four categories: (1) MM/MI (multimorbidity with mental illness)—concurrent presence of any mental illness and two chronic physical condition, (2) MM/No MI (multimorbidity)—concurrent presence of two or more chronic physical conditions but no mental illness, and (3) No MM—presence of only one physical illness but no mental illness.

### 2.6. Dependent Variable: Any Ambulatory Care Sensitive Hospitalizations (ACSHs)

We defined presence or absence of any ACSH based on the Agency for Healthcare Research and Quality (AHRQ) criteria [47]. ACSH indicators were developed by AHRQ after extensive reviews, testing, and validation. Investigators from Stanford University and University of California (on behalf of AHRQ) conducted structured and systematic review of existing literature in two phases (Phase I: 1994; Phase II: 1999–2001) to measure hospital quality. Overall, 14 indicators were selected and validated through face validity, precision testing, minimum bias, construct validity, fostering of quality improvement and applicability. These indicators were selected because they were highly sensitive to high-quality outpatient care. Indeed, findings from the systematic review had suggested a strong link between limited access to primary and coordinated outpatient care and hospitalizations for these 14 conditions [48]. AHRQ then developed composite Prevention Quality Indicators (PQIs) to generate a summary indicator which would help in measuring quality across multiple indicators and improve statistical power to detect quality differences [49]. We used AHRQ's PQI software to classify persons with any ACSH; ACSH for chronic conditions; ACSH for acute conditions.

Overall, any ACSHs include hospitalizations for any of the following 12 conditions. They are (1) diabetes short-term complications; (2) diabetes long-term complications; (3) COPD; (4) hypertension; (5) congestive heart failure; (6) dehydration; (7) bacterial pneumonia; (8) urinary infections; (9) angina without a procedure; (10) uncontrolled diabetes; (11) adult asthma; (12) lower extremity amputations. Medicare beneficiaries who had a hospitalization for any of the above-mentioned conditions in the observed calendar year were considered to have an ACSH. Conditions were identified by International Classification of Diseases, 9th edition, clinical modification (ICD-9-CM) codes [50]. 

### 2.7. Other Independent Variables

Demographic variables were gender (women and men), race/ethnicity (white, African American, Latino, and other), age in years (less than 55 years, 56–64, 65–69, 70–74, and 75 or older), marital status (married, widowed, divorced/separated, and other), metropolitan status (metro, nonmetro), and census region. Socioeconomic status included education (less than high school, high school, some college, and college), poverty status (less than 200% Federal Poverty Line (FPL) and greater than 200% FPL), Medicaid (yes and no), private insurance (yes and no), and prescription drug coverage (yes and no). Health status was measured by self-perceived general health (excellent, very good, good, fair, and poor), smoking status (current, past, and never) and body mass index (BMI) categories (underweight (0–18.5 kg/m^2^); normal (18.5–25.0 kg/m^2^); overweight (25.0–30.0 kg/m^2^), obese (30.0–40.0 kg/m^2^); morbidly obese (40.0 kg/m^2^ and above)). Functional status was based on a count of activities of daily living (ADL) limitations.

Prior literature on the determinants of ACSH has documented that coordinated care interventions improved care-continuity and reduced ACSH [51–54]. However, under the present organization of the Medicare fee-for-service system, seeking care from multiple providers almost always leads to fragmented care [55]. Those with MM/MI are particularly vulnerable because mental health care is often carved out of general healthcare system [56] and a reimbursement system that does not provide financial incentives for care coordination [57]. Therefore, we also controlled for whether the Medicare beneficiary was seeking care from multiple providers. We defined primary care provider use as having visited physicians practicing general internal medicine and family medicine. Specialist care provider use was defined based on visits to specialty providers such as oncologists, psychiatrists, and other specialties. Provider type was categorized into four groups: primary care provider use (PCP) only; specialist care provider use (SCP) only; both PCP/SCP; other. Year of panel was used as an additional covariate to control for time effects. All independent variables were measured at the baseline.

### 2.8. Statistical Methods

Subgroup differences in ACSH rates were determined by chi-square tests of independence. The relationship between different multimorbidity categories and any ACSH was examined with regression models in which independent variables were entered in blocks. Model I only examined the relationship between multimorbidity and ACSH while not controlling for any other independent variables. Model II controlled for year of observation, gender, race/ethnicity, age, marital status, metro status, region, supplemental insurance, self-perceived general health, functional status, smoking status, and BMI categories. In addition to those variables controlled for in Model II, we also included provider type in Model III.

In order to assess the differences between MM/MI and MM without mental illness groups, we also conducted regression models with MM without mental illness as the reference group. Results from both sets of analyses are presented. Due to the low prevalence of ACSH (<10%), we followed the recommendations of Zhang and Yu whereby adjusted odds ratios (AORs) approximate relative risk [58]. Therefore, AOR and relative risk of ACSH are used interchangeably. All analyses controlled for the complex sample design of MCBS and were conducted using survey procedures with Statistical Analysis System software (SAS version 9.2 Cary, NC, USA) [59].

## 3. Results

### 3.1. Description of the Study Sample

A majority of the Medicare beneficiaries studied were women, aged greater than 65 years old, and married. Most of the beneficiaries resided in a metropolitan area and had at least high school education.

### 3.2. Multimorbidity and Sample Characteristics

More than three-fourth of beneficiaries had multiple chronic conditions regardless of the presence of mental illness. Fourteen percent reported multimorbidity with concurrent mental illness (MM/MI). As shown in Table 1, MM/MI was significantly higher in women compared to men; Latinos compared to white; those aged less than 55 years old compared to 75 years and older; beneficiaries with income less 200% FPL compared to those with income greater than 200% FPL; current smokers compared to past smokers; morbidly obese individuals compared to beneficiaries with normal weight. Interestingly, rate of MM/MI was significantly higher among those who use multiple providers for their healthcare needs as compared to those who sought care from SCP or PCP only.

### 3.3. Multimorbidity and Any Preventable Hospitalization: Bivariate Relationships

In this study sample, the overall prevalence of ACSH was 8.1%. Among inpatient users the rate of ACSH was 25.1%. As shown in Table 2, percent with any ACSH was highest (10.8%) among Medicare beneficiaries with MM/MI, followed by those with MM without any mental illness (8.8%), and lowest among those with single chronic condition and no mental illness, that is, No MM group (1.1%). The differences in rates were statistically significant at *P* < 0.001. Comparison of any ACSH among multimorbidity groups suggested that the rate of any ACSH was higher among those with MM/MI compared to those with MM without any mental illness (Table 3, unadjusted odds ratios from Model I). Medicare beneficiaries with MM/MI were 30% more likely than those with MM and no mental illness to have any ACSH (OR = 1.30; 95%  CI = 1.05, 1.57). The same patterns were observed for any acute ACSH. We found that Medicare beneficiaries with MM/MI were more likely to have acute ACSH (defined as dehydration, bacterial pneumonia, and urinary infection) as compared to those with MM and no mental illness.

Among inpatient users, percent with any ACSH was highest (27.6%) among Medicare beneficiaries with MM/MI and those with MM and no mental illness (25.7%) and lowest among those with single chronic condition and no mental illness, that is, No MM group (19.1%). Similar patterns were observed for any chronic ACSH. Comparison between MM/MI and MM without any mental illness for acute ACSH revealed no statistically significant differences between these two groups.

### 3.4. Multimorbidity, Mental Illness, and ACSH: Multivariable Logistic Regressions

Adjusted odds ratios and 95% confidence intervals for multimorbidity categories from logistic regressions on any ACSH are presented in Table 3. The top panel displays results using the reference group No MM, whereas the bottom panel presents results using the reference group MM without any mental illness. As mentioned previously, these regressions controlled for independent variables in blocks: Model I did not adjust for any other independent variables; Model II adjusted for cohort variable, gender, race/ethnicity, age, marital status, metro status, education, Medicaid coverage, private insurance coverage, health status, functional status, body mass index, and smoking status; Model III additionally adjusted for provider type. In Model II, the likelihood of any ACSH was higher among Medicare beneficiaries with MM/MI (AOR = 1.87, 95%  CI = 1.32, 2.64) and MM without mental illness (AOR = 1.73, 95%  CI = 1.27, 2.38) as compared beneficiaries without multimorbidity, that is, No MM group. In Model III, after additionally controlling for provider type, likelihood of any ACSH was reduced for both groups (AOR = 1.62, 95%  CI = 1.14, 2.30 and AOR = 1.54, 95%  CI = 1.12, 2.11, resp.).

As seen from the bottom panel of Table 3, except for the unadjusted Model 1, we did not observe a significant association between ACSH and multimorbidity. For example, after adjusting for demographic, health status, functional status, and life-style risk factor variables there was not a statistically significant difference in the likelihood of any ACSH among beneficiaries with MM/MI as compared to those with MM without mental illness (AOR = 1.08, 95%  CI = 0.87, 1.34).

An interesting and noteworthy finding was the association between any ACSH and provider type. Seeking care from multiple provider types (i.e., both primary care and specialists) was associated with increased likelihood of any ACSH. Medicare beneficiaries seeking care from multiple providers were approximately three times as likely as those seeking care only from primary care providers to have any ACSH (AOR = 1.82, 95%  CI = 1.48, 2.23). It has to be noted that there was no significant difference in the likelihood of any ACSH between individuals seeking care from PCP only or SCP only.

## 4. Discussion

This paper was set out to examine the association between a special case of multimorbidity with mental illness and quality of care measured by ACSH. Among Medicare beneficiaries with at least one of the following seven physical chronic conditions (arthritis, cancer diabetes, heart diseases (myocardial infarction, coronary heart disease, and other heart conditions), hypertension, respiratory diseases (chronic obstructive pulmonary disease (COPD), and asthma), and osteoporosis), we found that three quarters of Medicare beneficiaries had multimorbidity. Of these with multimorbidity 18% had both mental illness and two chronic physical conditions. Approximately one-fifth of the Medicare beneficiaries reported having no multimorbidity. These findings support and confirm prior literature documenting the high prevalence of the conditions included in our study [43–45].

As expected, the rates of any ACSH were higher among Medicare beneficiaries with multimorbidity compared to those without multimorbidity. After controlling for other independent variables (panel, gender, race/ethnicity, age, marital status, metro status, education, Medicaid coverage, private insurance coverage, health status, functional status, body mass index, and smoking status), we did not observe statistically significant differences in ACSH between those with MM/MI and those with MM but no mental illness. Thus, despite complexities and challenges involved in the management of individuals with concurrent mental and chronic physical conditions, presence of mental illness did not indicate poor quality care as represented by any ACSH.

Furthermore, in multiple logistic regressions, with adjustment for provider type, the magnitude of the association between multimorbidity and any ACSH was somewhat reduced. We also found that provider type had an independent and statistically significant association with any ACSH. Seeking care from multiple providers (i.e., both primary care and specialist care providers) increased the likelihood of any ACSH. It is plausible that under the fee-for-service structure, seeking care from both primary care and specialty care providers may potentially lead to fragmentation in care. Indeed it has been suggested that under the existing financial incentives in the fee-for-service Medicare program, physicians often practice in isolated groups without sharing patient information with other providers [55, 57].

Although the data used in this study is USA specific, our findings have international implications because ACSHs are widely accepted quality of care indicators in many countries. For example, ACSHs also known as avoidable/preventable hospitalizations have been identified by the Organization for Economic Co-operation and Development (OECD) as important indicators for measuring health care quality [60]. Indeed, Gusmano and colleagues [61] have used ACSHs as a measure to compare health systems in Manhattan and Paris. Similarly, another study assessed the quality of Spain's ambulatory care systems using rates of ACSH for specific conditions (hypertension, asthma, and uncontrolled diabetes) [62]. Findings from these studies suggest that despite diverse health systems, access to primary care is a significant predictor of ACSH.

In addition, authors of the OECD report acknowledge that challenges related to poor care coordination or fragmentation of care are universal among individuals with multiple chronic conditions [60]. Our study findings on the link between fragmentation of care, multimorbidity, and preventable hospitalizations are applicable in any setting where fragmentation of care is an issue. Our findings can be also used to design programs to reduce the extent of fragmentation of care for individuals with multiple chronic conditions.

Furthermore, the study findings strengthen the argument in favor of developing healthcare delivery models specifically designed to coordinate care between providers in the USA. Recognizing the adverse effects of fragmented care, policy makers, and providers has placed increased emphasis on coordinated care delivery models such as Medical Home and Accountable Care Organizations. We know, for example, the Patient Protection and Affordable Care Act of 2010 calls for improvements in access to primary care and care coordination and includes preventable hospitalizations as a priority for quality improvement within the Medicare system [63].

This study has many strong points such as use of nationally representative data, longitudinal study design, adequate sample size due to pooling of multiple longitudinal panels, ability to assess prevention quality indicators, and use of many variables that may affect any ACSH. Linked Medicare claims and survey data are often described as “best of both worlds” [64] because they provide information on characteristics that are usually not found in claims data (e.g., functional status and general health status). There are, however, some limitations which should be considered when interpreting the findings. Some data elements are self-reported. Specifically, our identification of mental illness was based on self-reports. Although, MCBS followed special field procedures including interviewing respondents at relatively short intervals and verification of information (i.e., including examining explanation of benefits forms) to minimize recall bias, identification of mental illness with routinely collected survey data may be limiting. While we recognize the limitations of self-reported data for mental health conditions, it should be noted that routinely collected survey data are often used for mental health surveillance and assessing clinical, economic and social burden associated with mental illness in the USA [65]. Another limitation of the study was that the sample was restricted to only fee-for-service enrollees and cannot be generalized to the general Medicare population. Additionally, even though we pooled multiple survey panels sample sizes were too small to analyze specific types of ACSH.

Despite these limitations, this is the first study, to our knowledge, to examine the association between any ACSH and multimorbidity with and without mental illness. Findings from our study suggest that regardless of the presence of mental illness, multimorbidity is associated with any ACSH, and fragmented care could partially explain the increased likelihood of ACSH in this population. The emerging healthcare delivery models intended to reduce fragmentation of care may help in reducing the risk of preventable hospitalizations among Medicare beneficiaries with multimorbidity.

## Figures and Tables

**Table 1:** Description of study sample characteristics by multimorbidity categories among Medicare beneficiaries Medicare Current Beneficiary Survey, 2000–2005.

	Total		Multimorbidity categories			
			MM/MI	MM/No MI	No MM	Sig
	*N*	Wt%	Wt%	Wt%	Wt%	
ALL	8,963	100.0	13.8	66.5	19.7	***
Panels						***
2000–2002	2,244	24.0	8.8	71.6	19.6	
2001–2003	2,260	25.1	13.8	65.8	20.4	
2002–2004	2,217	24.7	16.1	65.0	19.0	
2003–2005	2,242	26.2	16.2	64.0	19.7	
Sex						***
Women	5,011	56.8	15.6	66.8	17.6	
Men	3,952	43.2	11.5	66.1	22.4	
Race/ethnicity						***
White	7,156	80.4	13.3	67.3	19.4	
African American	892	9.4	13.2	69.0	17.8	
Latino	563	6.3	20.0	56.7	23.3	
Others	339	3.9	16.0	61.3	22.7	
Age in years						***
Less than 55	778	6.1	44.2	37.1	18.7	
56–64	488	6.5	38.9	53.4	7.7	
65–69	1,816	26.1	10.1	64.1	25.8	
70–74	1,676	20.3	11.1	67.5	21.4	
75 and older	4,205	41.1	9.1	74.0	16.9	
Marital status						***
Married	4,745	55.9	11.6	67.2	21.3	
Widowed	2,791	28.9	12.1	72.5	15.4	
Divorced/separated	904	10.5	27.3	54.0	18.7	
Others	519	4.7	20.9	50.1	29.0	
Metro status						
Metro	6,002	71.2	13.8	65.9	20.3	
Not metro	2,960	28.8	13.9	68.0	18.0	
Education						***
No high school	2,875	30.0	15.9	66.8	17.3	
High school	3,251	36.7	13.4	67.6	19.0	
Some college	1,229	14.4	14.8	65.9	19.3	
College	1,578	18.9	10.5	64.7	24.8	
Poverty status						***
GT 200% FPL	4,107	49.0	9.9	67.6	22.5	
LT 200% FPL	4,856	51.0	17.5	65.5	17.0	
General health						***
Excellent	1,166	13.7	4.3	61.2	34.4	
Very good	2,318	27.0	7.4	65.6	27.1	
Good	2,896	32.1	11.5	71.9	16.6	
Fair	1,797	19.2	22.7	67.9	9.4	
Poor	747	8.0	39.2	54.8	5.9	
Functional status (ADL)						***
None	6,199	71.1	9.7	66.4	23.9	
1–3	1,924	20.7	20.6	69.3	10.1	
3 or more	825	8.2	31.8	61.3	6.9	
Body mass index						***
Underweight	201	2.1	12.2	64.2	23.6	
Normal	3,128	34.0	11.9	64.8	23.3	
Overweight	3,385	38.7	12.1	67.7	20.2	
Obese	1,900	22.2	17.9	67.4	14.7	
Morbid	283	3.0	29.1	64.2	6.7	
Smoking status						***
Current smoker	1,199	13.5	24.6	56.0	19.3	
Past smoker	4,145	47.0	12.4	69.1	18.5	
Never smoked	3,605	39.5	11.8	67.2	21.0	
Care coordination						***
PCP/SPEC	1,917	23.1	17.1	72.6	10.4	
SPEC only	917	11.0	14.0	72.0	14.0	
PCP only	3,024	36.2	13.2	68.1	18.7	
None	2,489	29.7	11.6	63.3	25.2	

Note: Based on 8,963 Medicare beneficiaries who were followed for 3 years and were first interviewed either in 2000, 2001, 2002, or 2003 and not enrolled in Medicare Health Maintenance organizations during the observation years. Asterisks represent significant group differences between multimorbidity categories and sample characteristics based on chi-square tests at *P* < 0.001.

ADL: activities of daily living; FPL: federal poverty line; GE: greater than or equal; Wt: weighted, MM/MI: multimorbidity with mental illness; MM/No MI: multimorbidity without mental illness; No MM: no multimorbidity or only one condition; PCP/SPEC: primary care provider and specialty care use; SPEC: specialist care use only; PCP: primary care provider use only.

**Table 2:** Number and weighted percent with ambulatory care sensitive hospitalizations by multimorbidity among Medicare beneficiaries Medicare current beneficiary survey, 2000–2005.

	MM/MI		MM/No MI		No MM		Sig
	*N*	Wt%	*N*	Wt%	*N*	Wt%
	Multimorbidity classification						

Any ACSH							***
Yes	139	10.8	578	8.8	72	4.0	
No	1,150	89.2	5,384	91.2	1,640	96.0	
Acute ACSH							**
Yes	62	4.4	254	3.7	44	2.4	
No	1,227	95.6	5,708	96.3	1,668	97.6	
Chronic ACSH							***
Yes	93	7.5	399	6.2	34	1.9	
No	1,196	92.5	5,563	93.8	1,678	98.1	

	Among inpatient users only						

Any ACSH							*
Yes	139	27.6	578	25.7	72	19.1	
No	381	72.4	1,586	74.3	305	80.9	
Acute ACSH							
Yes	62	11.3	254	10.9	44	11.5	
No	458	88.7	1,910	89.1	333	88.5	
Chronic ACSH							***
Yes	93	19.3	399	18.2	34	9.1	
No	427	80.7	1,765	81.8	343	90.9	

Note: Based on 8,963 Medicare beneficiaries who were followed for 3 years and were first interviewed either in 2000, 2001, 2002, or 2003 and not enrolled in Medicare Health Maintenance organizations during the observation years.

Asterisks represent significant group differences between multimorbidity categories and preventable hospitalizations based on chi-square tests.

ACSH: ambulatory care sensitive hospitalizations; Wt: weighted

***P < 0.001; **0.001 < *P* < 0.01; *0.01 < *P* < 0.05.

**Table 3:** Adjusted odds ratio and 95% confidence interval from logistic regression on any ambulatory care sensitive hospitalizations Medicare current beneficiary survey, 2000–2005.

ALL	Model I			Model II			Model III		
	OR	95% CI	Sig	AOR	95% CI	Sig	AOR	95% CI	Sig
Analysis I (Reference Group = No MM)									

Multimorbidity									
MM/MI	2.81	[2.03, 3.88]	***	1.87	[1.32, 2.64]	***	1.62	[1.14, 2.30]	**
MM/No MI	2.16	[1.59, 2.94]	***	1.73	[1.27, 2.38]	***	1.54	[1.12, 2.11]	**
**No MM**	Reference group			Reference group			Reference group		

Analysis II (Reference Group = MM/No MI)									

Multimorbidity									
MM/MI	1.30	[1.08, 1.57]	**	1.08	[0.87, 1.34]		1.05	[0.84, 1.31]	
**MM/No MI**	Reference group			Reference group			Reference group		
No MM	0.46	[0.34, 0.63]	***	0.58	[0.42, 0.79]	***	0.65	[0.47, 0.89]	**

Note: Analytic sample consists of 8,963 Medicare beneficiaries who were followed for 3 years (described as panels) and were first interviewed either in 2000, 2001, 2002, or 2003 and not enrolled in Medicare Health Maintenance organizations during the observation years.

The logistic regressions also include intercept terms not presented here. Asterisks represent significant group differences compared to the reference group based on logistic regressions on any ambulatory care sensitive hospitalizations.

Model I: logistic regressions only controlled for multimorbidity categories.

Model II: logistic regressions additionally controlled for panel, gender, race/ethnicity, age, marital status, metro status, education, Medicaid coverage, private insurance coverage, health status, functional status, body mass index, and smoking status.

Model III: logistic regressions additionally controlled for provider-type variable along with all the variables included in Model II.

***P < 0.001; **0.001 < *P* < 0.01; *0.01 < *P* < 0.05.
